# Halitosis: A Prospective Cohort Study Comparing Periodontal Debridement with Tongue Cleaning, Denture Biofilm Management, and Restorative Maintenance

**DOI:** 10.3390/dj14090595

**Published:** 2026-09-14

**Authors:** Adrian Boloș, Otilia Cornelia Boloș, Edida Maghet, Romina Georgiana Bita, Raluca Briceag, Bogdan Andrei Bumbu, Silviu Brad

**Affiliations:** 1 Department of Oral Rehabilitation, Faculty of Dental Medicine, Specialization of Dental Technology, “Victor Babeș” University of Medicine and Pharmacy, 300041 Timișoara, Romania; bolos.adrian@umft.ro; 2 Department of Dental Aesthetics, Faculty of Dental Medicine, “Victor Babeș” University of Medicine and Pharmacy, 300041 Timișoara, Romania; bolos.otilia@umft.ro; 3 Faculty of Dental Medicine, “Victor Babeș” University of Medicine and Pharmacy, 300041 Timișoara, Romania; edida.maghet@umft.ro; 4 2nd Department, Radiology and Medical Imaging, General and Dento-Maxillary Imaging, Faculty of Dental Medicine, “Victor Babeș” University of Medicine and Pharmacy, 300041 Timișoara, Romania; brad.silviu@umft.ro; 5 Faculty of Dental Medicine, Ovidius University of Constanța, 900684 Constanța, Romania; 6 Department of Dental Medicine, Faculty of Medicine and Pharmacy, University of Oradea, 410073 Oradea, Romania; bogdanbumbu@uoradea.ro

**Keywords:** halitosis, aged, dentures, oral hygiene, periodontal debridement

## Abstract

**Background:** Halitosis in elderly dental patients is rarely attributable to a single etiology; tongue coating, periodontal inflammation, denture biofilm, and reduced salivary flow may coexist and modify treatment response. We compared three procedure pathways and examined whether tongue-coating reduction mediates procedure-related improvement. **Methods:** A prospective cohort of 94 patients aged 65 years or older was assigned by dominant indication to periodontal debridement with tongue cleaning (PDT, *n* = 33), denture biofilm management (DBM, *n* = 31), or restorative/prosthetic maintenance (RPM, *n* = 30). This was a single-center, nonrandomized prospective cohort study; assignment followed the dominant clinical indication rather than randomization. All clinical assessments were made by a single examiner who was masked to the formal procedure-group assignment; masking was partial because the clinical picture at follow-up could sometimes reveal the care delivered. The primary outcome was percentage reduction in volatile sulfur compound (VSC) concentration at 8 weeks. Secondary outcomes included organoleptic score, HALT, OHIP-14, tongue-coating index, denture plaque index, and a composite clinical response (≥30% VSC reduction plus ≥ 1-point organoleptic improvement plus ≥ 8-point HALT improvement). Linear mixed-effects, multivariable regression, and bootstrap mediation analyses were performed. **Results:** All three procedures reduced VSC at 8 weeks, but PDT produced the largest absolute reduction (−102.6 ± 20.2 ppb) and the highest composite response rate (57.6%), versus 9.7% in DBM and 3.3% in RPM (*p* < 0.001). Adjusted analyses showed PDT improved VSC reduction by 22.8 percentage points and DBM by 11.7 percentage points relative to RPM. Tongue-coating reduction mediated 29.5% of the PDT effect and 35.6% of the DBM effect. Xerostomia and reduced salivary flow attenuated the response in DBM and RPM but not in PDT. **Conclusions:** Periodontal debridement with tongue cleaning was the most effective procedure pathway for elderly halitosis. Tongue-coating reduction was a meaningful, though statistically rather than experimentally established, mediator of treatment benefit, supporting an oral-ecological framework for managing geriatric oral malodor that warrants confirmation in randomized studies.

## 1. Introduction

Halitosis is a common oral-health complaint with both biological and psychosocial consequences. In most patients, oral malodor is generated by anaerobic bacterial metabolism of sulfur-containing substrates, resulting in volatile sulfur compounds such as hydrogen sulfide, methyl mercaptan, and dimethyl sulfide [1,2,3,4]. Although transient malodor can follow pungent foods, clinically relevant halitosis is frequently sustained by tongue coating, periodontal inflammation, poor oral hygiene, and reduced oral clearance [3,4,5,6]. These mechanisms are particularly important in older adults, in whom retained plaque, gingival inflammation, prosthetic surfaces, and medication-related dryness may coexist. Work published since 2020 has continued to refine the understanding of halitosis as a microbially driven, multifactorial condition and has reinforced the role of the tongue dorsum and periodontal niches as principal intra-oral sources, while also highlighting the contribution of removable prostheses in older populations [7,8,9,10].

Elderly dental patients represent a distinct population for halitosis research because oral malodor is rarely attributable to one isolated factor. Aging is commonly accompanied by tooth loss, removable prosthetic rehabilitation, root caries, periodontal pocketing, mucosal fragility, polypharmacy, and reduced salivary reserve [11,12,13,14]. In this context, halitosis can become a marker of broader oral ecological imbalance rather than a single symptom. Moreover, the social effects of malodor may be amplified in older adults who already face barriers to nutrition, social interaction, denture confidence, and oral-health-related quality of life.

Removable dentures introduce additional plaque-retentive surfaces that may support bacterial and fungal biofilm. Denture surfaces can accumulate food debris and microbial deposits, particularly when patients have reduced manual dexterity, poor prosthesis fit, overnight denture wear, or inconsistent cleaning routines [11,12,15]. Previous work in prosthetic dentistry has suggested that denture hygiene and prosthesis-related factors may contribute to malodor, yet denture-focused halitosis studies often do not simultaneously measure tongue coating, salivary flow, and patient-reported outcomes. This limits clinical interpretation because denture biofilm may influence breath odor indirectly through changes in the tongue and mucosal environment.

Periodontal disease is another major pathway to persistent oral malodor. Periodontal pockets create anaerobic niches in which proteolytic bacteria degrade amino acids and release volatile sulfur compounds [4,7,16]. Bleeding on probing, increased probing depth, tongue coating, and elevated VSC values are therefore biologically connected. In older adults, periodontal debridement may reduce both subgingival reservoirs and inflammatory substrate availability, while adjunctive tongue cleaning may address the dorsal tongue as a major malodor source. A procedure that targets both reservoirs may therefore be expected to outperform dental maintenance procedures that do not directly disrupt these biofilms.

Xerostomia and reduced salivary flow add another layer of complexity. Saliva supports oral clearance, buffering, lubrication, swallowing, and antimicrobial defense [13,14,17,18]. When salivary flow is reduced, desquamated cells, food debris, and bacterial substrates may persist on the tongue, gingival margin, and prosthetic surfaces. Dry-mouth symptoms may also impair denture comfort and increase patient awareness of unpleasant taste or breath. Therefore, salivary-flow phenotype may modify treatment response, especially when interventions rely primarily on hygiene reinforcement rather than direct mechanical biofilm disruption.

The primary objective was to compare halitosis outcomes after three procedure pathways: periodontal debridement with tongue cleaning, denture biofilm management, and restorative/prosthetic maintenance. Secondary objectives were to assess whether xerostomia, salivary-flow status, tongue-coating reduction, denture plaque, and prosthesis fit modified response. We hypothesized that periodontal debridement with tongue cleaning would produce the greatest VSC and organoleptic improvement, and that tongue-coating reduction would partly mediate procedure-related halitosis improvement.

## 2. Materials and Methods

### 2.1. Study Design and Participants

This study used a prospective cohort design. The cohort included 94 elderly dental patients aged 65 years or older who presented consecutively to a university-affiliated dental clinic with self-reported or clinician-suspected oral malodor and who met the eligibility criteria; all were prospectively recruited and assessed by the study team. To remove any ambiguity about the study population, we clarify that all 94 participants were drawn from a single source population—outpatients aged 65 years or older attending this university-affiliated dental clinic for routine or problem-focused dental care during the recruitment period, in whom oral malodor was either self-reported or clinically suspected—and were enrolled consecutively as they satisfied the eligibility criteria detailed in Section 2.2. The three procedure groups were not separate populations but mutually exclusive subsets of this same cohort, distinguished only by the dominant dental indication identified at baseline. When more than one relevant indication was present, the patient was assigned to the group corresponding to the clinically dominant problem, as judged by the treating clinician before any outcome assessment; this single-center sampling frame also delimits the population to which the findings apply. The observation period included baseline, 2-week, and 8-week assessments. The three procedure pathways were selected to represent common geriatric dental scenarios in which halitosis may be driven by periodontal biofilm, removable denture biofilm, or restorative/prosthetic maintenance needs. The study protocol was approved by the Ethics Committee for Scientific Research of “Victor Babeș” University of Medicine and Pharmacy, Timișoara, Romania (approval E-787/8 February 2023) and conducted in accordance with the Declaration of Helsinki. Because the three procedures could not be concealed from the patients or from the operators who delivered them, the study was conducted as an open-label cohort with masked outcome assessment. The extent of that masking, and its limits, are specified in Section 2.3 and revisited in Section 4.2; the same terminology is used consistently throughout the manuscript.

### 2.2. Procedure Groups and Geriatric Oral-Health Classification

Patients were assigned to one of three procedure groups according to their dominant dental indication. The periodontal debridement plus tongue cleaning group (PDT, *n* = 33) represented patients with periodontal inflammation, tongue-coating accumulation, and malodor judged to be primarily biofilm-related. The denture biofilm management group (DBM, *n* = 31) represented patients whose dominant issue was removable denture plaque, prosthesis hygiene difficulty, or ill-fitting prosthetic surfaces. The restorative/prosthetic maintenance group (RPM, *n* = 30) represented patients requiring crown polishing, restoration adjustment, prosthetic maintenance, or hygiene reinforcement without a targeted periodontal debridement or denture disinfection protocol. Because patients were allocated according to their dominant dental indication rather than by randomization, the three groups represent distinct baseline oral phenotypes, each receiving the standard care indicated for its dominant problem. The study should therefore be interpreted as an observation of how different baseline pathologies respond to their respective, pathway-appropriate treatment rather than as a head-to-head comparison of three interventions applied to a single, common condition; statistical adjustment can attenuate but cannot remove the resulting between-group non-comparability, and cannot substitute for randomization.

The inclusion criteria were age at least 65 years, ability to complete questionnaires, and completion of baseline and 8-week measurements. Exclusion criteria included recent systemic antibiotic use, acute odontogenic infection requiring emergency treatment, recent oral surgery, active oral malignancy, severe cognitive impairment preventing questionnaire completion, and inability to cooperate with organoleptic evaluation. Additional geriatric variables included denture use, ill-fitting prosthesis, polypharmacy, Charlson comorbidity index, xerostomia status, unstimulated salivary flow, tongue-coating index, denture plaque index among denture users, mean probing pocket depth, and bleeding on probing.

### 2.3. Outcome Measures and Follow-Up Assessments

The primary outcome was percentage reduction in volatile sulfur compound concentration at 8 weeks. VSC values were measured in parts per billion using a portable chairside sulfide monitor (Halimeter, Interscan Corp., Chatsworth, CA, USA), which is most sensitive to hydrogen sulfide and methyl mercaptan and reports a single summed VSC value in ppb; it does not separately resolve dimethyl sulfide. The monitor was calibrated against the manufacturer’s reference standard before each measurement session. To control for the diurnal variation in oral malodor, all measurements were obtained at the same time of day (mid-morning), and patients were instructed to refrain from eating, drinking, smoking, toothbrushing, and the use of toothpaste, mouthrinse, or other oral-hygiene aids for at least 2 h before assessment. Two consecutive readings were obtained at each visit (approximately 1 min apart, each preceded by a 30 s period of mouth closure), and their mean was used for analysis; the absolute difference between duplicate readings was required to fall within the device’s reported repeatability; otherwise, a third reading was taken. Secondary outcomes included an organoleptic score from 0 to 5, a Halitosis Associated Life-Quality Test (HALT) total score from 0 to 100, an Oral Health Impact Profile-14 (OHIP-14) score from 0 to 56 [15], a tongue-coating index from 0 to 12, a denture plaque index, and a composite clinical response. The tongue-coating index was scored using the Winkel tongue coating index (WTCI) [19], in which the tongue dorsum is divided into six sextants, each scored 0–2 for coating, giving a total range of 0–12. The denture plaque index among denture wearers was scored using the index described by Augsburger and Elahi, rating the fitting surface of the prosthesis from 0 (no plaque) to 4 (heavy plaque) [20]. All clinical assessments (VSC, organoleptic scoring, periodontal probing, and tongue-coating scoring) were performed by a single examiner who did not deliver any of the study interventions and who was masked to each patient’s formal procedure-group assignment; intra-examiner reliability, assessed on repeated examinations of a subset of participants, exceeded an intraclass correlation coefficient (or weighted kappa) of 0.80 for each measure. The organoleptic score is acknowledged to retain a subjective component even under masked conditions. We further acknowledge that complete masking of the assessor with respect to the procedure pathway could not always be maintained throughout follow-up because the residual clinical picture at re-examination—for example, evidence of recent periodontal debridement, professional denture cleaning, or restorative/prosthetic maintenance—could in some cases reveal, or allow inference of, the treatment a patient had received. This limitation applies principally to the subjective organoleptic score and, to a lesser extent, to the other examiner-rated clinical measures, whereas the instrument-based VSC readings are largely unaffected by knowledge of group allocation. The masking described here should therefore be understood as masking of the formal procedure-group label rather than as guaranteed masking of the treatment actually delivered. To keep this description internally consistent, the examiner is referred to throughout the manuscript as partially masked rather than as blinded, and the corresponding statements in the Results, Discussion, and Study Limitations sections have been aligned with this definition. Composite response required three simultaneous improvements: at least 30% VSC reduction, at least 1-point organoleptic improvement, and at least 8-point HALT improvement. These composite thresholds were specified a priori to approximate minimal clinically important differences (MCIDs) for each domain: a 30% reduction is a commonly used clinically meaningful change for VSC in halitosis studies, a 1-point shift on the 0–5 organoleptic scale represents a perceptible change in examiner-rated malodor, and an 8-point change on the HALT corresponds to approximately half of one standard deviation of the baseline HALT distribution in this cohort, a recognized distribution-based estimate of an MCID. We acknowledge that formally validated, instrument-specific MCIDs for the HALT and for subjective organoleptic scales are not firmly established in the literature, and that the chosen thresholds therefore remain partly pragmatic. The supplementary “VSC < 150 ppb” endpoint used a 150 ppb cut-off because this approximates the level above which oral malodor becomes organoleptically detectable to a typical examiner with this class of monitor.

Baseline assessments were performed before the procedure, with VSC and organoleptic reassessment at 2 weeks and full reassessment at 8 weeks. The PDT intervention consisted of full-mouth periodontal debridement with standardized tongue-cleaning instruction. The DBM intervention consisted of professional denture cleaning, denture plaque disclosure, prosthesis hygiene counseling, and adjustment of obvious plaque-retentive prosthetic irregularities. The RPM intervention consisted of restorative polishing, prosthetic adjustment, and general oral-hygiene reinforcement. Before each assessment, patients avoided food, alcohol, smoking, mouthwash, and oral-hygiene aids for the standardized pre-assessment interval described above.

### 2.4. Statistical Analysis

Continuous variables were summarized as mean ± standard deviation, and categorical variables were summarized as number and percentage. Between-group comparisons used one-way analysis of variance for continuous variables and chi-square tests for categorical variables. Within-group baseline-to-follow-up changes were examined using paired *t*-tests. The distributional assumptions underlying these parametric tests were checked before they were applied. Normality of the continuous variables and of the within-patient change scores was assessed with the Shapiro–Wilk test together with visual inspection of histograms and normal quantile–quantile plots, and homogeneity of variance across the three procedure groups was assessed with Levene’s test. Volatile sulfur compound (VSC) concentration, VSC change, HALT score, OHIP-14 score, unstimulated salivary flow, mean probing pocket depth, and bleeding on probing were compatible with normality (Shapiro–Wilk *p* > 0.05) and with homoscedasticity (Levene *p* > 0.05), so one-way ANOVA and paired *t*-tests were retained for these variables; where normality held but variances were unequal, the Welch correction was applied. The ordinal measures—organoleptic score, Winkel tongue-coating index, and denture plaque index—did not satisfy these assumptions, and for these variables the Kruskal–Wallis test with Dunn post hoc comparisons replaced one-way ANOVA and the Wilcoxon signed-rank test replaced the paired *t*-test. The non-parametric results agreed with the parametric results in both direction and statistical significance, and the means and standard deviations are therefore retained in the results tables for comparability with the other outcomes. For the categorical comparisons, expected cell counts were inspected before the chi-square approximation was used, and Fisher’s exact test was substituted wherever an expected count fell below five, which applied to the composite-response endpoint and to several subgroup cells. Correlation analyses used Spearman’s rho because several clinical measures were ordinal or not guaranteed to follow a normal distribution. Statistical significance was set at *p* < 0.05.

To provide a more comprehensive statistical framework, repeated VSC measurements at baseline, 2 weeks, and 8 weeks were analyzed using a linear mixed-effects model with patient identifier as the grouping variable. Time was entered as a categorical factor (baseline, 2 weeks, 8 weeks) so that no linear time trend was imposed on the trajectory; a random intercept for patient accounted for the within-patient correlation of the repeated measurements, and the model was fitted by restricted maximum likelihood. This specification does not require the sphericity assumption of repeated-measures ANOVA. Model adequacy was examined by plotting the conditional residuals against the fitted values to detect non-constant variance and by normal quantile–quantile plots of both the conditional residuals and the estimated random intercepts; neither indicated a material departure from the assumptions of the model. Because every patient contributed measurements at all three time points, the analysis was complete by construction, and no imputation of missing data was required. A multivariable linear regression model was then used to identify independent predictors of 8-week percentage VSC reduction. Procedure group, tongue-coating reduction, xerostomia, salivary flow, baseline VSC, prosthesis fit, age, and sex were included as predictors. The assumptions of this model were examined by plotting the residuals against the fitted values to assess linearity and homoscedasticity, by a normal quantile–quantile plot of the residuals, and by the Breusch–Pagan test; multicollinearity was quantified with variance inflation factors, all of which were below 2.5, and influential observations were screened with Cook’s distance, no value of which exceeded 0.5. With eight predictors and 94 observations, approximately 12 observations were available per estimated parameter, which we treated as the maximum model complexity that this sample size could support; no further covariates or interaction terms were therefore fitted. Finally, a bootstrap mediation analysis estimated how much of the procedure’s effect on VSC reduction was mediated through tongue-coating reduction. The mediation model was an adjusted model: the mediator (tongue-coating reduction) and outcome (8-week percentage VSC reduction) regressions both included the same covariates as the multivariable model (baseline VSC, xerostomia, salivary flow, prosthesis fit, age, and sex), so that the reported indirect and direct effects are conditional on these covariates rather than unadjusted. Bias-corrected and accelerated bootstrap confidence intervals for the indirect effect were obtained from 2000 resamples, and the approach to causal mediation followed the framework of Imai and colleagues [21] and Hayes [22]. Identification of an indirect effect in a nonrandomized design rests on the sequential-ignorability assumption, that is, on the absence of unmeasured confounding of the mediator–outcome relationship. Because this assumption cannot be verified from the data, the robustness of the indirect effect was examined using the residual correlation sensitivity analysis of Imai and colleagues [21], in which the correlation (ρ) between the error terms of the mediator and outcome models is varied systematically and the value of ρ at which the estimated indirect effect crosses zero is recorded. The bootstrap procedure itself imposes no distributional assumption on the sampling distribution of the indirect effect, which is typically skewed, and bias-corrected and accelerated intervals were therefore preferred to normal-theory intervals. For the longitudinal outcomes, between-group differences in 8-week change scores were compared across the three procedure groups using one-way ANOVA (or the Kruskal–Wallis test where distributional assumptions were not met), with Bonferroni-corrected post hoc pairwise comparisons. Only the primary outcome, the 8-week percentage reduction in VSC, was pre-specified for confirmatory inference; the *p*-values reported for the secondary outcomes, correlations, and subgroup analyses were not adjusted for the number of comparisons performed and should be read as descriptive and exploratory. ROC curves for predicting composite response were compared using the DeLong method for correlated areas under the curve, with AUC values interpreted using conventional benchmarks (approximately 0.7–0.8 acceptable, 0.8–0.9 excellent) following Hosmer and Lemeshow [23], and 95% confidence intervals reported for each AUC. The sample size was not based on a formal a priori power calculation; the cohort size was determined by the number of eligible patients who presented consecutively during the recruitment period. To characterize the resolution of the design rather than to justify it retrospectively, a sensitivity calculation was performed in which the smallest difference detectable with 80% power at a two-sided alpha of 0.05 was derived for group sizes of 30–33 from the observed between-patient variability in VSC reduction. This minimum detectable difference was approximately 12 percentage points, rising to approximately 22–25 percentage points for the smallest subgroup cells (*n* = 10–12). We emphasize that this is a design-based sensitivity calculation and not an estimate of achieved power. Post hoc or observed power computed from the effect size actually obtained is a deterministic function of the *p*-value and therefore conveys no information beyond it, so it cannot be used to argue that a study was adequately powered or that a non-significant result reflects insufficient power [24]. The calculation above is only presented to indicate the magnitude of effect that this cohort was able to resolve, and the non-significant findings, in particular the null covariate coefficients in the multivariable model and the wide subgroup estimates, should be read as inconclusive rather than as evidence of the absence of an effect. Analyses were conducted in R version 4.3, using the lme4, mediation, pROC, and boot packages.

## 3. Results

Demographic and clinical characteristics by procedure group are summarized in Table 1. The three groups were comparable in age, sex distribution, Charlson comorbidity index, polypharmacy, and unstimulated salivary flow (all *p* > 0.05). Significant differences were observed in characteristics that defined the procedure pathway: current denture use was universal in DBM (100.0%) versus 42.4% in PDT and 43.3% in RPM (*p* < 0.001); ill-fitting prostheses were more frequent in DBM (51.6%, *p* = 0.012); and self-reported xerostomia was highest in DBM (67.7%) and lowest in RPM (33.3%, *p* = 0.026). These imbalances are consistent with the indication-driven group assignment and were addressed in the multivariable models.

Baseline halitosis, oral ecology, and quality-of-life measures by group are presented in Table 2. DBM patients entered the study with the highest baseline VSC concentration (250.7 ± 37.6 ppb), organoleptic score (3.6 ± 0.6), HALT total score (61.4 ± 8.3), and tongue-coating index (8.9 ± 1.1), whereas PDT patients had the highest mean probing pocket depth (4.5 ± 0.8 mm) and bleeding on probing (33.3 ± 7.4%; all *p* < 0.001). RPM patients had the mildest baseline halitosis profile across most measures. These baseline patterns reflect the underlying clinical phenotypes that motivated each procedure pathway.

Within-group changes from baseline to 2 weeks and 8 weeks are shown in Table 3. All three groups demonstrated statistically significant improvements across every halitosis and quality-of-life outcome (within-group *p* < 0.05). The magnitude of improvement, however, differed substantially: PDT achieved the largest VSC reduction (−102.6 ± 20.2 ppb), organoleptic improvement (−1.1 ± 0.3 points), HALT decrease (−14.7 ± 4.8 points), and OHIP-14 decrease (−6.4 ± 3.2 points). DBM produced intermediate improvements, while RPM yielded the smallest changes—particularly for organoleptic score (−0.3 ± 0.5) and OHIP-14 (−1.6 ± 2.7). Formal between-group comparison of the 8-week change scores confirmed that these differences were statistically significant: one-way ANOVA across the three groups was significant for VSC change, organoleptic change, HALT change, and OHIP-14 change (all *p* < 0.001). Bonferroni-corrected post hoc tests showed that PDT produced significantly greater improvement than both DBM and RPM on every outcome (all adjusted *p* < 0.01), and that DBM produced significantly greater improvement than RPM for VSC and HALT change (adjusted *p* < 0.05). For the organoleptic score in particular, the PDT change (−1.1) was significantly larger than the RPM change (−0.3; adjusted *p* < 0.001).

Binary response endpoints at 8 weeks are reported in Table 4. PDT achieved the highest rate on every endpoint: ≥30% VSC reduction in 90.9% (vs. 45.2% in DBM and 23.3% in RPM), organoleptic improvement ≥ 1 point in 60.6% (vs. 12.9% and 10.0%), and HALT improvement ≥ 8 points in 93.9% (vs. 71.0% and 23.3%; all *p* < 0.001). The composite response, requiring all three endpoints simultaneously, was achieved by 57.6% of PDT patients compared with 9.7% of DBM and 3.3% of RPM patients (*p* < 0.001), indicating that meaningful multi-domain improvement was overwhelmingly concentrated in the PDT pathway.

Subgroup analyses by xerostomia and salivary-flow phenotype are shown in Table 5. PDT consistently outperformed the other procedures in every subgroup (all *p* < 0.001 within subgroup). VSC reduction in PDT remained substantial even among patients with xerostomia (39.1 ± 7.6%) and reduced salivary flow (39.1 ± 6.2%), although less than in patients with normal salivary status. In contrast, DBM and especially RPM showed marked attenuation of response in dry-mouth subgroups, with RPM yielding only 9.2% mean VSC reduction in xerostomic patients and no composite responders. These data suggest that PDT is the most robust choice when salivary impairment is present. Of note, within the PDT pathway, the composite response rate was numerically higher in xerostomic patients (11/16, 68.8%) than in non-xerostomic patients (8/17, 47.1%), a direction opposite to that seen in DBM and RPM. This apparent paradox is most plausibly explained by confounding by baseline severity: xerostomic PDT patients entered with higher baseline VSC and more pronounced tongue coating, leaving greater room for absolute and composite improvement once subgingival and tongue reservoirs were mechanically disrupted, whereas the percentage VSC reduction (the continuous measure) remained lower in the same xerostomic subgroup. Because the relevant cell sizes are small (*n* = 16 and *n* = 17), this observation should be regarded as hypothesis-generating rather than definitive.

Spearman correlations between selected baseline and outcome variables are presented in Table 6. Baseline VSC correlated very strongly with the organoleptic score (ρ = +0.86) and HALT score (ρ = +0.83), and inversely with salivary flow (ρ = −0.71; all *p* < 0.001), supporting concurrent validity across biochemical, clinical, and patient-reported domains. Tongue coating correlated moderately with both baseline VSC (ρ = +0.53) and organoleptic score (ρ = +0.49), reinforcing its role as a major malodor reservoir. Higher baseline OHIP-14 score showed a weak inverse association with 8-week VSC reduction (ρ = −0.22, *p* = 0.035), suggesting that more severe baseline impact may modestly limit proportional improvement.

The linear mixed-effects model for repeated VSC measurements is summarized in Table 7. The PDT × 8-week interaction was the largest negative coefficient (β = −69.7 ppb, 95% CI −79.4 to −60.1), confirming that PDT produced the steepest VSC trajectory beyond what would be expected from the RPM-referenced time effect. The DBM × 8-week interaction (β = −35.7 ppb) was about half as large. Self-reported xerostomia was independently associated with higher VSC (β = +29.4 ppb, *p* = 0.001), and each 1.0 mL/min increment in salivary flow was associated with a 234.6 ppb decrease in VSC (*p* < 0.001). Age was not significant (*p* = 0.746).

Independent predictors of percentage VSC reduction at 8 weeks are reported in Table 8. After adjustment for clinical and demographic covariates, PDT was associated with a 22.8 percentage-point greater VSC reduction than RPM (95% CI +14.4 to +31.2; *p* < 0.001) and DBM with an 11.7 percentage-point greater reduction (*p* = 0.002). Each 1-point reduction in tongue-coating index was associated with a further 4.5 percentage-point improvement in VSC reduction (*p* = 0.005). Higher baseline VSC slightly attenuated proportional reduction (β = −0.1; *p* = 0.010). Xerostomia, salivary flow, prosthesis fit, age, and sex were not significant after adjustment, and the model explained 65.8% of the variance.

Bootstrap mediation analysis quantifying how much of the procedure’s effect on VSC reduction is transmitted through tongue-coating reduction is shown in Table 9. For PDT versus RPM, the total effect of +32.3% decomposed into a direct effect of +22.8% and an indirect effect via tongue coating of +9.5%, corresponding to 29.5% mediation. For DBM versus RPM, the total effect of +18.2% comprised a direct effect of +11.7% and an indirect effect of +6.5%, with 35.6% mediation. All effects were statistically significant. These results identify tongue-coating reduction as a meaningful, partially mediating mechanism, while substantial direct procedure’s effects remain—particularly for PDT, where periodontal-pocket disruption likely contributes independently. The indirect and direct effects reported here are derived from a covariate-adjusted mediation model (adjusting for baseline VSC, xerostomia, salivary flow, prosthesis fit, age, and sex). Because the design was observational and nonrandomized and the mediator was not experimentally manipulated, these estimates represent a statistical decomposition of association rather than experimentally established causal mediation. In the sensitivity analysis, the estimated indirect effect fell to zero at a mediator–outcome residual correlation of ρ ≈ 0.32 for the PDT contrast and ρ ≈ 0.36 for the DBM contrast. An unmeasured factor acting on both tongue-coating reduction and VSC reduction with a residual correlation of about this magnitude would therefore be sufficient to account for the whole of the mediated pathway, so the mediation estimates are best described as moderately, rather than strongly, robust.

**Figure 1:**
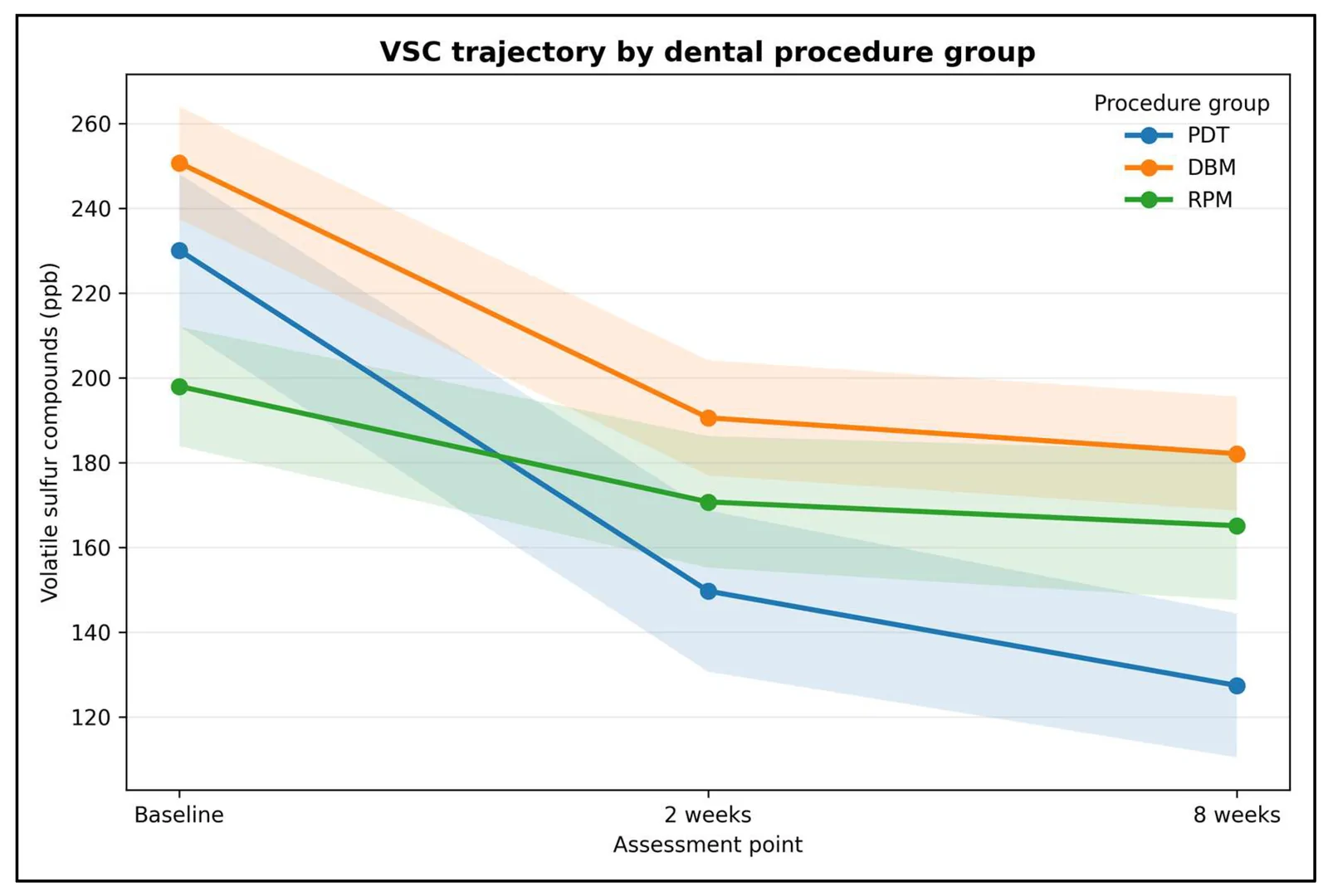
VSC trajectory by dental procedure group. Lines show the group means VSC concentration at each assessment; shaded bands represent the 95% confidence interval of the mean. All patients contributed measurements at every time point (PDT *n* = 33, DBM *n* = 31, RPM *n* = 30 at baseline, 2 weeks, and 8 weeks); no participants were lost to follow-up.

Figure 1 shows the mean VSC concentration trajectory at baseline, 2 weeks, and 8 weeks for each procedure group. All groups demonstrated reductions over time, but the trajectories diverged markedly: PDT showed the steepest and most sustained decline, DBM showed an intermediate decline that plateaued between 2 and 8 weeks, and RPM showed the smallest reduction throughout follow-up. The visual pattern is consistent with the mixed-effects model coefficients in Table 7.

**Figure 2:**
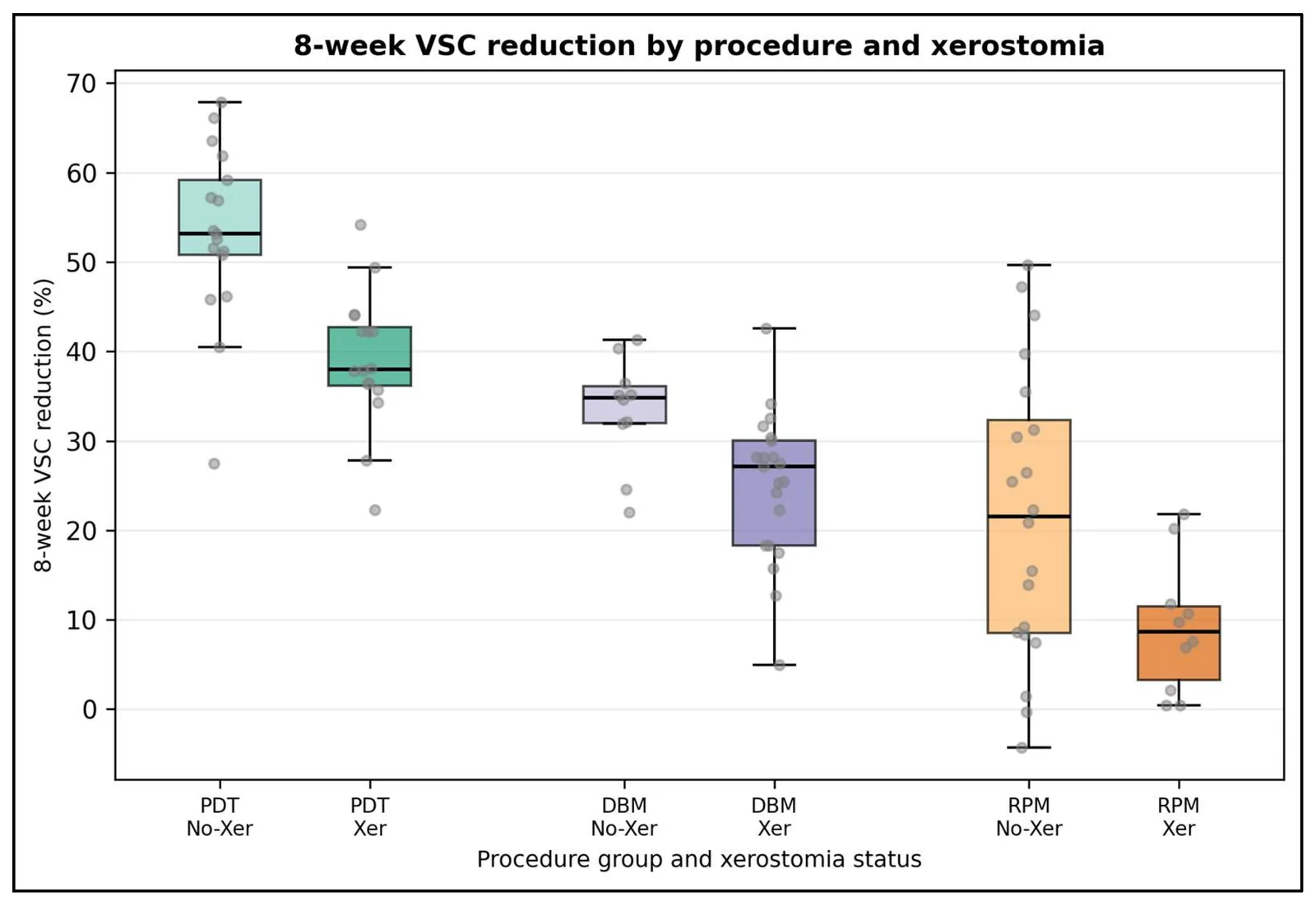
Distribution of 8-week VSC reduction by procedure group and xerostomia. Boxes show the median and interquartile range; whiskers extend to 1.5 times the interquartile range, with individual points overlaid. For the continuous percentage reduction outcome shown here, xerostomic patients had lower reductions than non-xerostomic patients within every procedure group, including PDT; this is distinct from the composite-response rate (Table 5), which was higher in xerostomic PDT patients owing to their greater baseline severity (see Results and Discussion).

Figure 2 displays the distribution of 8-week percentage VSC reduction by procedure group, stratified by xerostomia status. PDT achieved the highest median reduction in both subgroups, with relatively narrow dispersion. DBM and RPM showed lower medians and were more sensitive to xerostomia, with the xerostomic boxes shifted toward smaller reductions. The visual pattern corroborates the subgroup analyses in Table 5 and reinforces that PDT is the most robust pathway under salivary impairment.

**Figure 3:**
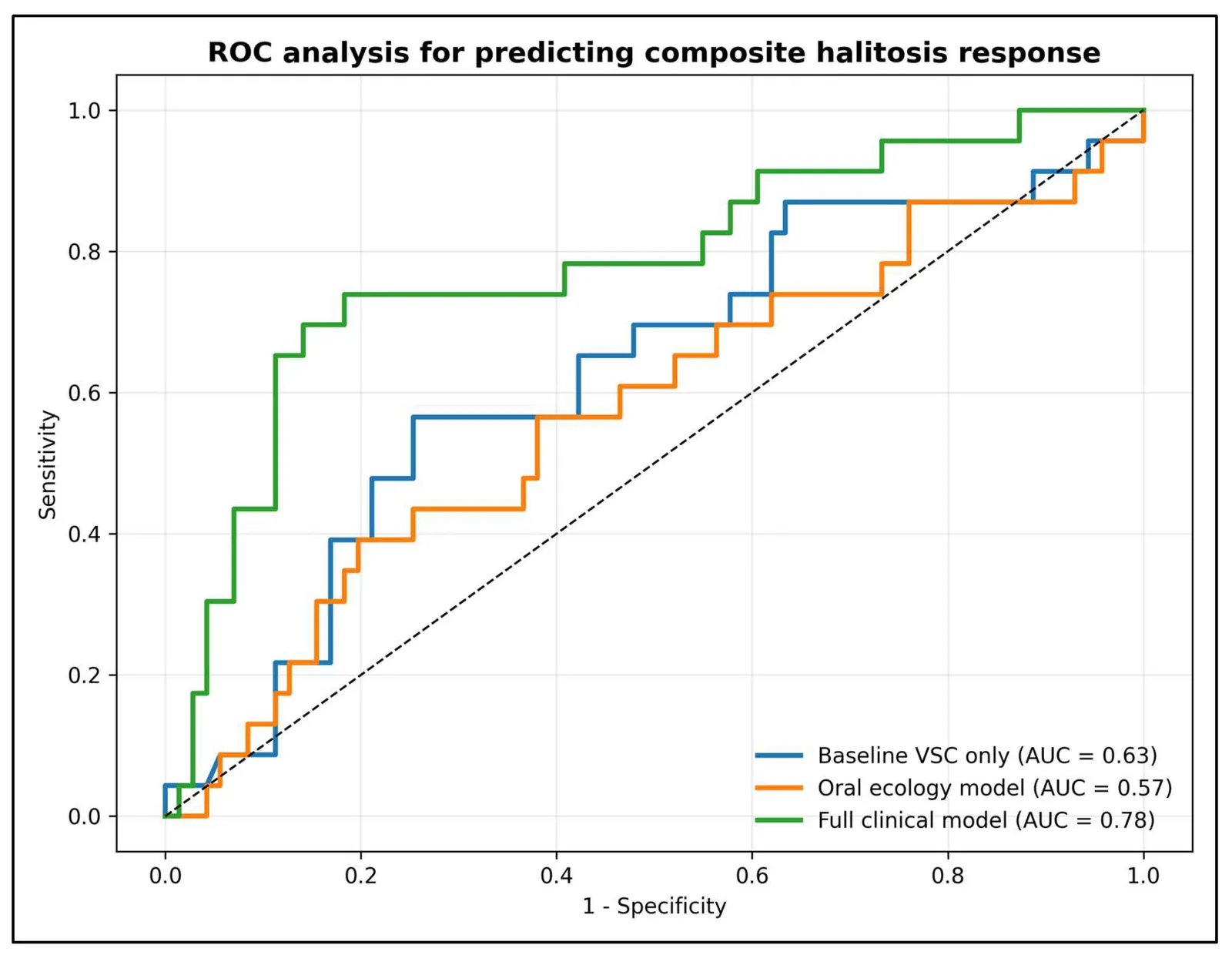
ROC analysis for predicting composite halitosis response. AUC (95% CI): baseline VSC only 0.63 (0.51–0.75); oral-ecology model 0.57 (0.45–0.69); full clinical model 0.78 (0.68–0.88). Pairwise DeLong tests: full vs. oral-ecology *p* = 0.012; full vs. baseline-VSC *p* = 0.061; baseline-VSC vs. oral-ecology *p* = 0.42.

Figure 3 presents the receiver-operating characteristic (ROC) analysis for predicting the composite halitosis response. The model combining procedure assignment with key oral-ecology covariates provides good discrimination between responders and non-responders, supporting the prognostic value of these baseline variables when integrated with the procedure pathway. Sensitivity and specificity at clinically meaningful thresholds can be read directly from the curve. The full clinical model achieved acceptable-to-excellent discrimination (AUC = 0.78, 95% CI 0.68–0.88), outperforming both the baseline-VSC-only model (AUC = 0.63, 95% CI 0.51–0.75) and the oral-ecology-only model (AUC = 0.57, 95% CI 0.45–0.69). Pairwise DeLong tests indicated that the full model significantly outperformed the oral-ecology model (*p* = 0.012) and showed a non-significant trend toward outperforming the baseline-VSC model (*p* = 0.061), while the baseline-VSC and oral-ecology models did not differ significantly from each other (*p* = 0.42). The observation that the oral-ecology model alone discriminated slightly worse than baseline VSC alone likely reflects that, in isolation, the oral-ecology variables (tongue coating, salivary flow, denture status) are correlated proxies for the same underlying malodor burden captured more directly by baseline VSC, and add incremental discrimination only when combined with procedure assignment in the full model. Given the wide and overlapping confidence intervals, these AUC differences should be interpreted cautiously.

## 4. Discussion

### 4.1. Analysis of Findings

This study suggests that elderly patients with halitosis may respond differently depending on whether the dominant clinical pathway is periodontal/tongue biofilm, denture biofilm, or restorative/prosthetic maintenance. This interpretation is consistent with the prior literature describing oral malodor as a multifactorial condition driven by microbial degradation, tongue coating, periodontal inflammation, and prosthesis-associated reservoirs [8,25,26,27,28]. In this cohort, PDT produced the largest VSC reduction, the greatest organoleptic improvement, and the highest composite response rate. DBM also improved biochemical malodor, but weaker organoleptic and composite responses suggest that denture cleaning alone may not fully resolve malodor when dry mouth, prosthesis fit, and tongue coating remain active. These findings support conceptualizing elderly halitosis as an oral-ecological syndrome rather than as a single-procedure outcome. The OHIP-14 results deserve specific interpretation, as patient-reported quality of life is a key dimension of halitosis management. PDT produced a mean OHIP-14 reduction of 6.4 points, compared with 3.9 points for DBM and only 1.6 points for RPM. On a 0–56 scale, a 6-point improvement represents a clinically perceptible reduction in the oral-health-related quality-of-life burden (roughly an 11% improvement relative to the scale range and approximately one standard deviation of the baseline OHIP-14 distribution), whereas the 1.6-point RPM change, although statistically significant, is unlikely to be perceived by patients as meaningful. The OHIP-14 trajectory therefore parallels the biochemical and organoleptic findings and reinforces that the benefit of PDT extends beyond breath chemistry into patient-perceived function and comfort. It should also be acknowledged that better-resourced published trials of periodontal debridement (for example, Quirynen et al. [16]) and of antimicrobial mouthrinse regimens (Dadamio et al. [29]) incorporated microbiological characterization and longer follow-up than the present study, and direct comparison with that work is limited by the absence of microbiological data and the 8-week horizon here.

The most clinically interesting finding is the mediation effect of tongue-coating reduction. In the present data, tongue-coating improvement explained a meaningful proportion of the benefit from both PDT and DBM. This finding extends the study beyond a simple comparison of dental procedures because the tongue dorsum has been repeatedly identified as a major malodor reservoir and a target for mechanical and antimicrobial interventions [19,29,30,31,32]. The persistent direct effect of PDT suggests that periodontal debridement contributes independently by reducing subgingival anaerobic niches, bleeding, and proteolytic substrate availability. Conversely, the DBM effect appeared more dependent on tongue-coating reduction, suggesting that denture biofilm control may improve breath partly by reducing microbial transfer from prosthetic surfaces to mucosal and tongue reservoirs.

This mediation result nevertheless requires cautious interpretation because it was obtained in an observational cohort in which neither the procedure nor the mediator was experimentally manipulated. A mediation model identifies an indirect effect only under sequential ignorability, which requires both that group assignment be independent of the potential outcomes given the measured covariates and that no unmeasured factor confounds the mediator–outcome relationship. Neither condition is guaranteed in an indication-driven cohort, and the sensitivity analysis reported above shows that a residual mediator–outcome correlation of approximately 0.32–0.36 would be enough to reduce the indirect effect to zero; oral-hygiene compliance, manual dexterity, and patient motivation are plausible candidates for such a factor and were not measured here. A second constraint is temporal. The mediator and the outcome were both recorded at the 8-week visit, so the causal ordering between them is imposed by the model rather than established by the sequence of measurement, and a reciprocal pathway in which improved breath reinforces adherence to tongue cleaning, and thereby reduces coating, cannot be excluded. Third, only one mediator was modeled, whereas bleeding on probing, denture plaque, and salivary function are all correlated with tongue coating and may transmit part of the same effect; the proportion mediated is consequently better read as the share of the association traveling through a tongue-coating pathway that also carries these correlated processes than as the isolated contribution of the coating itself. Any measurement error in the tongue-coating index would in addition attenuate the mediator–outcome path, and the proportion-mediated statistic is itself imprecisely estimated at this sample size. We therefore regard the estimates of 29.5% and 35.6% as an internally consistent and biologically coherent decomposition that generates a testable hypothesis, namely that mechanical reduction in tongue coating is an active component of the treatment effect, rather than as evidence that tongue-coating reduction causes the improvement. A direct test would require randomization of the tongue-cleaning component itself, for example, a factorial trial in which periodontal debridement is delivered with and without adjunctive tongue cleaning, with the mediator measured at an interim visit that precedes outcome assessment.

The subgroup findings also emphasize the relevance of salivary-flow phenotype. Low salivary flow reduced proportional VSC improvement, especially in DBM and RPM patients, while PDT maintained a stronger response. This aligns with the literature describing saliva as a key determinant of oral clearance, mucosal lubrication, antimicrobial defense, denture comfort, and patient-reported oral function [33,34,35,36,37]. Xerostomia does not render halitosis untreatable, but it may require repeated mechanical biofilm control, salivary stimulation strategies, prosthesis adjustment, and reinforced hygiene follow-up. For future clinical research, these findings support prospective designs that stratify older adults by xerostomia, denture status, tongue coating, periodontal burden, and oral-health-related quality of life rather than treating geriatric halitosis as a homogeneous entity.

### 4.2. Study Limitations and Shortcomings

The principal shortcomings of this study are its single-center setting, nonrandomized indication-driven group assignment, short 8-week follow-up, partial assessor masking, absence of microbiological characterization, and limited statistical power for exploratory subgroup and mediation analyses. This study has several limitations that should be considered when interpreting the findings. The cohort was recruited at a single university-affiliated center, and the indication-driven assignment to procedure pathway introduced baseline imbalances that, although addressed in mixed-effects and multivariable models, cannot fully replace randomization. We emphasize that statistical adjustment can reduce, but cannot eliminate, confounding arising from a nonrandomized design, and cannot reproduce the baseline comparability that randomization would confer; accordingly, the adjusted between-pathway estimates are best read as describing how distinct baseline pathologies responded to their respective standard care rather than as an unbiased causal comparison of interchangeable treatments for the same condition. Because assignment was nonrandomized, unmeasured confounders—such as patient motivation, hygiene-compliance history, denture age, the specific medications responsible for xerostomia, and frequency of prior dental care—cannot be excluded, and the adjusted estimates from the multivariable and mediation models therefore cannot be interpreted as establishing causation. For the mediation analysis in particular, the sequential-ignorability assumption cannot be tested, the mediator and the outcome were measured concurrently at 8 weeks, and only a single mediator was modeled; the sensitivity analysis indicated that an unmeasured mediator–outcome confounder of moderate strength would suffice to nullify the indirect effect, and the proportion mediated should accordingly be treated as a hypothesis for testing in a randomized factorial design. The 8-week follow-up captures short-term response but does not address durability of benefit, recurrence of biofilm reservoirs, or long-term effects on oral-health-related quality of life. This horizon is also shorter than the minimum 12-week follow-up recommended by the European Federation of Periodontology for reporting periodontal debridement outcomes; because the PDT VSC trajectory in Figure 1 was still declining between 2 and 8 weeks without a clear plateau, the 8-week measurement may underestimate the eventual treatment effect, and relapse kinetics beyond 8 weeks remain unknown. VSC measurement was based on a chairside sulfide monitor that is sensitive to hydrogen sulfide and methyl mercaptan but does not separately resolve dimethyl sulfide and other extra-oral contributions, and organoleptic scoring, although standardized, retains a subjective component. Assessments were performed by a single, partially masked examiner, as defined in Section 2.3, which ensured internal consistency but precluded estimation of inter-examiner reliability; future studies should employ several calibrated examiners masked to group allocation and report both intra- and inter-examiner reliability. In addition, although the examiner was masked to the formal group allocation, masking with respect to the procedure pathway itself could not be guaranteed because the clinical status observed at follow-up may have revealed the type of care a patient had received; this residual risk of partial assessor unmasking is most relevant to subjective outcomes such as the organoleptic score, and the possibility that it introduced some degree of bias into the examiner-rated measures cannot be excluded. Microbiological characterization of subgingival, tongue-dorsum, and denture biofilms was not performed, limiting mechanistic conclusions about specific bacterial shifts. In the absence of microbiological data, statements about microbial mechanisms are presented as biologically plausible inferences rather than direct observation, and the corresponding claims have been framed cautiously. Finally, the sample of 94 patients restricts power for higher-order interactions and rare-subgroup estimates, and external validity to non-university and non-European geriatric dental populations should be confirmed in future prospective work, ideally through randomized, adequately powered, multicenter studies. In this connection, we note explicitly that no a priori sample-size calculation was performed and that the sensitivity calculation reported in Section 2.4 should not be read as a demonstration of adequate power, since power estimated after the fact from the observed effect adds nothing to the *p*-value already reported [24]. The cohort is best regarded as adequately sized for the primary between-pathway contrast in VSC reduction and as underpowered for the subgroup, interaction, and mediation analyses, which are correspondingly reported as exploratory.

## 5. Conclusions

In this prospective cohort of elderly dental patients, periodontal debridement combined with tongue cleaning was the most effective of the three procedure pathways, producing the largest reduction in volatile sulfur compounds, the greatest organoleptic and quality-of-life improvement, and the highest composite response rate. Denture biofilm management generated meaningful but more modest biochemical improvement, and its benefit was more sensitive to dry-mouth status. Tongue-coating reduction mediated approximately 30–36% of the procedure’s effect on VSC reduction, identifying the tongue dorsum as a clinically actionable target across procedure pathways. Because assignment was nonrandomized, this mediation result should be read as a statistical decomposition and a hypothesis of partial mediation rather than as established causal mediation. Xerostomia and reduced salivary flow attenuated response in the denture and restorative pathways but not appreciably in PDT, suggesting that targeted mechanical biofilm disruption is the most robust strategy when salivary reserve is limited. Taken together, these findings support the view that geriatric halitosis is best approached as a multifactorial oral-ecological condition. We suggest, as a direction for future prospective and adequately powered randomized studies with multiple examiners masked to group allocation rather than as a definitive practice recommendation, that routine assessment in older adults might usefully include tongue coating, periodontal status, denture hygiene, and salivary phenotype, and that treatments simultaneously addressing subgingival and tongue reservoirs warrant further evaluation.

## Figures and Tables

**Table 1:** Demographic and clinical characteristics by procedure group.

Variable	PDT (*n* = 33)	DBM (*n* = 31)	RPM (*n* = 30)	*p*-Value
Age, years	75.6 ± 5.9	74.7 ± 6.2	73.5 ± 5.3	0.372
Female sex	14 (42.4)	15 (48.4)	16 (53.3)	0.686
Charlson comorbidity index	3.3 ± 0.9	3.4 ± 0.9	3.5 ± 1.0	0.673
Polypharmacy, ≥5 medications	17 (51.5)	15 (48.4)	16 (53.3)	0.926
Current removable denture use	14 (42.4)	31 (100.0)	13 (43.3)	<0.001
Ill-fitting prosthesis	6 (18.2)	16 (51.6)	8 (26.7)	0.012
Self-reported xerostomia	16 (48.5)	21 (67.7)	10 (33.3)	0.026
Unstimulated salivary flow, mL/min	0.2 ± 0.1	0.2 ± 0.1	0.2 ± 0.1	0.251

Tests: one-way ANOVA for continuous variables and chi-square tests for categorical variables. All baseline characteristics were ascertained before procedure-group assignment; the between-group differences therefore reflect the indication-driven nature of the assignment rather than any effect of the procedures.

**Table 2:** Baseline halitosis, oral ecology, and quality-of-life measures.

Baseline Variable	PDT (*n* = 33)	DBM (*n* = 31)	RPM (*n* = 30)	*p*-Value
VSC concentration, ppb	230.0 ± 52.7	250.7 ± 37.6	198.0 ± 39.3	<0.001
Organoleptic score, 0–5	3.3 ± 0.8	3.6 ± 0.6	2.9 ± 0.8	<0.001
HALT total score, 0–100	55.2 ± 10.7	61.4 ± 8.3	51.4 ± 8.1	<0.001
OHIP-14 total score, 0–56	22.6 ± 5.6	23.7 ± 4.5	20.3 ± 5.6	0.045
Tongue coating index, 0–12	7.5 ± 1.4	8.9 ± 1.1	7.1 ± 1.6	<0.001
Mean probing pocket depth, mm	4.5 ± 0.8	3.5 ± 0.7	3.3 ± 0.5	<0.001
Bleeding on probing, % sites	33.3 ± 7.4	23.9 ± 6.8	18.9 ± 6.9	<0.001
Denture plaque index among denture users	2.7 ± 0.4	3.2 ± 0.6	2.7 ± 0.8	0.018

Tests: one-way ANOVA. Denture plaque was summarized only among denture users. The denture plaque index followed the Augsburger and Elahi scoring system (range 0–4) [20]; its *p*-value reflects a one-way ANOVA restricted to denture users only (PDT *n* = 14, DBM *n* = 31, RPM *n* = 13), so the denominators differ from the other rows.

**Table 3:** Baseline, 2-week, and 8-week changes in halitosis and quality-of-life outcomes.

Outcome	Group	Baseline	2 Weeks	8 Weeks	8-Week Change	Within-Group *p*
VSC concentration, ppb	PDT	230.0 ± 52.7	149.7 ± 55.7	127.4 ± 49.7	−102.6 ± 20.2	<0.001
VSC concentration, ppb	DBM	250.7 ± 37.6	190.5 ± 38.6	182.1 ± 38.2	−68.6 ± 19.7	<0.001
VSC concentration, ppb	RPM	198.0 ± 39.3	170.7 ± 43.3	165.1 ± 49.1	−32.9 ± 26.0	<0.001
Organoleptic score	PDT	3.3 ± 0.8	2.5 ± 0.9	2.2 ± 0.7	−1.1 ± 0.3	<0.001
Organoleptic score	DBM	3.6 ± 0.6	3.2 ± 0.5	3.0 ± 0.6	−0.6 ± 0.3	<0.001
Organoleptic score	RPM	2.9 ± 0.8	2.7 ± 0.8	2.6 ± 0.9	−0.3 ± 0.5	<0.001
HALT score	PDT	55.2 ± 10.7	—	40.5 ± 11.9	−14.7 ± 4.8	<0.001
HALT score	DBM	61.4 ± 8.3	—	50.4 ± 10.5	−11.0 ± 5.9	<0.001
HALT score	RPM	51.4 ± 8.1	—	47.2 ± 9.8	−4.3 ± 5.1	<0.001
OHIP-14 score	PDT	22.6 ± 5.6	—	16.2 ± 6.3	−6.4 ± 3.2	<0.001
OHIP-14 score	DBM	23.7 ± 4.5	—	19.7 ± 5.8	−3.9 ± 3.1	<0.001
OHIP-14 score	RPM	20.3 ± 5.6	—	18.7 ± 6.8	−1.6 ± 2.7	0.003

Within-group *p*-values were calculated using paired *t*-tests comparing baseline and 8-week values. Dashes (—) indicate that HALT and OHIP-14 were assessed only at baseline and 8 weeks and not at the 2-week visit, at which only VSC and organoleptic score were reassessed. Between-group differences in 8-week change scores were significant for all outcomes by one-way ANOVA (all *p* < 0.001); see text for Bonferroni-corrected pairwise comparisons.

**Table 4:** Clinical response endpoints at 8 weeks.

Response Endpoint	PDT (*n* = 33)	DBM (*n* = 31)	RPM (*n* = 30)	*p*-Value
≥30% VSC reduction	30 (90.9)	14 (45.2)	7 (23.3)	<0.001
Organoleptic improvement ≥ 1 point	20 (60.6)	4 (12.9)	3 (10.0)	<0.001
HALT improvement ≥ 8 points	31 (93.9)	22 (71.0)	7 (23.3)	<0.001
VSC < 150 ppb at 8 weeks	19 (57.6)	8 (25.8)	12 (40.0)	0.035
Composite clinical response	19 (57.6)	3 (9.7)	1 (3.3)	<0.001

Tests: chi-square tests. Composite response required ≥ 30% VSC reduction, ≥1-point organoleptic improvement, and ≥8-point HALT improvement.

**Table 5:** Subgroup analyses by xerostomia and salivary-flow phenotype. Each cell shows *n*; VSC reduction %, mean ± SD; composite response *n* (%).

Subgroup	PDT	DBM	RPM	*p*-Value
No xerostomia	17; 53.3 ± 9.9; 8 (47.1)	10; 33.4 ± 6.1; 1 (10.0)	20; 21.6 ± 16.3; 1 (5.0)	<0.001
Xerostomia	16; 39.1 ± 7.6; 11 (68.8)	21; 25.0 ± 8.3; 2 (9.5)	10; 9.2 ± 7.5; 0 (0.0)	<0.001
Salivary flow ≥ 0.18 mL/min	18; 52.5 ± 11.1; 8 (44.4)	19; 29.3 ± 9.6; 1 (5.3)	20; 20.5 ± 16.7; 1 (5.0)	<0.001
Salivary flow < 0.18 mL/min	15; 39.1 ± 6.2; 11 (73.3)	12; 25.2 ± 6.0; 2 (16.7)	10; 11.4 ± 9.1; 0 (0.0)	<0.001

Tests: one-way ANOVA within each subgroup. VSC = volatile sulfur compounds.

**Table 6:** Spearman correlation matrix for selected baseline and response variables.

Variable Pair	Spearman ρ	*p*-Value
Baseline VSC vs. Tongue coating	+0.53	<0.001
Baseline VSC vs. Salivary flow	−0.71	<0.001
Baseline VSC vs. Organoleptic score	+0.86	<0.001
Baseline VSC vs. HALT score	+0.83	<0.001
Baseline VSC vs. OHIP-14 score	+0.66	<0.001
Tongue coating vs. Organoleptic score	+0.49	<0.001
Salivary flow vs. Xerostomia inventory	−0.47	<0.001
Salivary flow vs. Organoleptic score	−0.67	<0.001
Organoleptic score vs. HALT score	+0.73	<0.001
HALT score vs. OHIP-14 score	+0.55	<0.001
OHIP-14 score vs. 8-week VSC reduction %	−0.22	0.035

Spearman correlation coefficients were used for all pairs.

**Table 7:** Linear mixed-effects model for repeated VSC concentration at baseline, 2 weeks, and 8 weeks.

Fixed Effect in Mixed Model	β for VSC ppb	95% CI	*p*-Value
2 weeks vs. baseline, RPM reference	−27.2	−34.2 to −20.3	<0.001
8 weeks vs. baseline, RPM reference	−32.9	−39.8 to −25.9	<0.001
DBM vs. RPM at baseline	+36.0	+18.8 to +53.1	<0.001
PDT vs. RPM at baseline	+20.3	+3.8 to +36.8	0.016
DBM × 2 weeks	−32.9	−42.6 to −23.1	<0.001
DBM × 8 weeks	−35.7	−45.5 to −25.9	<0.001
PDT × 2 weeks	−53.1	−62.7 to −43.5	<0.001
PDT × 8 weeks	−69.7	−79.4 to −60.1	<0.001
Self-reported xerostomia	+29.4	+11.5 to +47.3	0.001
Salivary flow, per 1.0 mL/min	−234.6	−341.1 to −128.1	<0.001
Age, per year	−0.2	−1.3 to +0.9	0.746

Reference category: RPM at baseline. Patient identifier was included as a grouping variable.

**Table 8:** Multivariable linear regression predicting 8-week VSC reduction percentage.

Predictor of 8-Week VSC Reduction (%)	Adjusted β	95% CI	*p*-Value
DBM vs. RPM	+11.7	+4.3 to +19.1	0.002
PDT vs. RPM	+22.8	+14.4 to +31.2	<0.001
Tongue coating reduction, per point	+4.5	+1.4 to +7.5	0.005
Self-reported xerostomia	−6.2	−12.7 to +0.2	0.059
Salivary flow, per 1.0 mL/min	−13.4	−54.0 to +27.3	0.515
Baseline VSC, per ppb	−0.1	−0.2 to −0.0	0.010
Ill-fitting prosthesis	−2.7	−7.3 to +1.9	0.250
Age, per year	+0.0	−0.4 to +0.4	0.963
Female sex	−1.1	−5.3 to +3.0	0.594

Adjusted R^2^ = 0.658. Reference category: RPM.

**Table 9:** Bootstrap mediation analysis: proportion of procedure’s effect mediated by tongue-coating reduction.

Contrast	Total Effect, % (95% CI)	*p* Total	Direct Effect, % (95% CI)	*p* Direct	Indirect Effect via Tongue, % (95% CI)	*p* Indirect	Proportion Mediated
DBM vs. RPM	+18.2 (+11.9 to +24.4)	<0.001	+11.7 (+4.3 to +19.1)	0.002	+6.5 (+2.5 to +11.8)	<0.001	35.6%
PDT vs. RPM	+32.3 (+26.8 to +37.8)	<0.001	+22.8 (+14.4 to +31.2)	<0.001	+9.5 (+3.6 to +17.5)	<0.001	29.5%

Bootstrap indirect effects were estimated with 2000 resamples; reference category: RPM.

## Data Availability

The raw data supporting the conclusions of this article will be made available by the authors on request.
